# Reviewed article: Ribeiro LF; Moura ED; Guimarães PC; Rosa TA. Completeness and clinical-epidemiological profile of notifications of venomous animal envenomations: a descriptive analysis in the Centro-Sul region of the State of Rio de Janeiro, 2020-2023. Epidemiol Serv Saude. 2026:35;e20250541

**DOI:** 10.1590/S2237-96222026v35e20250541.b

**Published:** 2026-11-09

**Authors:** William Meschial

**Affiliations:** 1 Universidade do Estado de Santa Catarina, Florianópolis, SC, Brazil Universidade do Estado de Santa Catarina Florianópolis SC Brazil

## Peer review B

### Questionnaire

Does the manuscript contain new and significant information to justify publication? Yes 

Does the Abstract (Summary) clearly and accurately describe the content of the article? No

Is the problem significant and concisely stated? No

Are the methods described comprehensively? No

Are the interpretations and conclusions justified by the results? No

Is adequate reference made to other work in the field? Yes 

### Manuscript Structure:

Length of article is: Adequate

Number of tables is: Adequate

Number of figures is: Adequate

### Rating

Interest Good

Quality Below Average

Originality Good

Overall Below Average

Recommendation: **Reject**

## Comments

Agradeço a oportunidade de revisar este manuscrito. A temática dos acidentes por animais peçonhentos e a análise da completude das fichas de notificação são de inegável importância para a saúde pública no Brasil, especialmente considerando os desafios na produção e distribuição de soros. No entanto, percebo que o manuscrito, tal como se apresenta, possui algumas fragilidades que precisam ser endereçadas para que sua progressão em um processo editorial.

Observo que o trabalho traz um achado particularmente interessante e de grande relevância que é a análise da indicação da soroterapia. A constatação de que o soro foi administrado em casos leves, contrariando as diretrizes do Ministério da Saúde e em um contexto de dificuldade de produção desses insumos estratégicos, é um ponto que merece profunda atenção e pode ser o grande diferencial do presente estudo. Encorajo-os a investir nessa análise, investigando com maior profundidade as razões e implicações dessas indicações equivocadas, o que poderia, inclusive, redefinir o foco central do artigo.

Ao analisar os resultados apresentados, parece-me que o objetivo principal do trabalho se inclina mais para a análise do perfil clínico e epidemiológico dos pacientes vítimas de acidentes por animais peçonhentos e para a avaliação da soroterapia, com a questão do preenchimento inadequado das fichas de notificação surgindo como um achado secundário, embora crucial. Essa possível desalinhamento entre o foco declarado e o achado de maior peso merece ser reconsiderado.

A representatividade dos dados é um ponto que demanda atenção. Embora Três Rios seja um polo para administração de soros, a coleta de dados restrita a uma única Unidade de Pronto Atendimento (UPA) em um município, aliada a um recorte temporal de três anos e meio que exclui importantes meses de verão (quando os acidentes são mais incidentes), limita a capacidade de generalização dos resultados. Um estudo descritivo com estas características locais e temporais pode ter dificuldade em refletir um cenário mais amplo ou completo, impactando a robustez das conclusões.

Para que o manuscrito ganhe a robustez necessária, sugiro as seguintes abordagens:

- Se o interesse primordial for o perfil clínico-epidemiológico dos acidentes e a avaliação da soroterapia, seria prudente ampliar o recorte temporal e considerar a exclusão de fichas com dados ausentes nas análises de perfil, reconhecendo esta ausência como uma limitação inerente da fonte de dados, mas não o foco principal. Isso permitiria uma análise mais consistente do perfil e da aplicação do soro.
- Caso a análise do preenchimento das fichas seja o objetivo central, o estudo ganharia imensa força ao complementar a análise quantitativa com uma abordagem qualitativa com os profissionais de saúde para compreender as razões da incompletude ou divergências nas informações. A análise conjunta com prontuários também poderia verificar não apenas a ausência de dados, mas a consistência e acurácia das informações registradas.
- Ainda, conforme mencionado, a avaliação da indicação da soroterapia é de grande valor. Sugiro que essa análise seja expandida, utilizando o prontuário como fonte primária para cruzar informações e avaliar a real indicação da soroterapia em cada caso, para além do que está nas fichas de notificação.

Após essas considerações, trago algumas análises direcionadas às seções do artigo.

Título e Resumo: o título é um tanto extenso e poderia ser mais direto ao refletir o objetivo central do trabalho. Uma sugestão, alinhando-se ao que percebo ser um forte diferencial, seria: “Aspectos clínicos e epidemiológicos de acidentes por animais peçonhentos e a adequação da soroterapia: estudo transversal. No resumo, é fundamental detalhar mais a metodologia, incluindo o tipo de análise realizada e o período exato de coleta de dados.

Introdução: a introdução precisa de um encadeamento mais fluido entre os parágrafos. Atualmente, a transição entre ideias pode ser aprimorada, garantindo mais coesão e lógica. A referência à problemática da subnotificação em 2008 (linhas 32-35) faria sentido apenas se acompanhada de referências mais atuais que demonstrem a persistência do problema. Isso reforça a relevância contínua da investigação sem depender de dados temporalmente distantes para ilustrar uma realidade atual.

Métodos: aqui é necessário melhorar substancialmente o detalhamento, justificando o recorte temporal de janeiro de 2020 a agosto de 2023. Quais foram os critérios de inclusão e exclusão dos dados? Os dados foram coletados de todo o universo de pacientes atendidos ou houve alguma seleção? É essencial descrever claramente quem realizou a coleta dos dados, como e quando ela ocorreu (qual o período de coleta, não apenas o período dos dados), e quais foram os procedimentos de análise de dados (software, testes estatísticos, abordagem para dados faltantes, etc.).

Resultados e Discussão: embora os resultados sejam apresentados de forma clara, a discussão deve aprofundar-se nas implicações da incompletude dos dados para a gestão e as políticas públicas. Além de constatar a falha, é importante explorar como essa ausência afeta, por exemplo, a alocação de recursos, a avaliação de impacto das intervenções e o planejamento de capacitações. A discussão sobre o uso inadequado da soroterapia é um ponto forte e deve ser ampliada, conectando-a com a problemática da produção de soros no país e suas consequências para o SUS.

Conclusão: a conclusão deve ser concisa e, idealmente, responder diretamente ao objetivo (reformulado, se for o caso), sem introduzir informações novas. Deve sintetizar os principais achados e suas implicações.

O potencial deste trabalho é considerável, e as questões que levanta são de grande valia para a comunidade científica e para a gestão em saúde pública. Contudo, para que atinja seu pleno potencial de impacto e seja aceito em uma revista de alto padrão é necessário um esforço considerável de revisão e aprofundamento.

